# Long-term usage of a commercial mHealth app: A “multiple-lives” perspective

**DOI:** 10.3389/fpubh.2022.914433

**Published:** 2022-11-10

**Authors:** Erica Y. Lau, Marc S. Mitchell, Guy Faulkner

**Affiliations:** ^1^Department of Emergency Medicine, University of British Columbia, Vancouver, BC, Canada; ^2^Vancouver Costal Health Research Centre, Centre for Clinical Epidemiology and Evaluation, Vancouver, BC, Canada; ^3^Faculty of Health Sciences, School of Kinesiology, Western University, London, ON, Canada; ^4^Population and Physical Activity Laboratory, School of Kinesiology, University of British Columbia, Vancouver, BC, Canada

**Keywords:** physical activity, mHealth, sustainability, implementation, usage patterns, user engagement

## Abstract

**Background:**

Emerging evidence suggests that individuals use mHealth apps in multiple disjointed ways in the real-world—individuals, for example, may engage, take breaks, and re-engage with these apps. To our knowledge, very few studies have adopted this ‘multiple-live’ perspective to analyze long-term usage of a physical activity (PA) app. This study aimed to examine the duration of use, as well as the frequency, length, and timing of streaks (uninterrupted periods of use) and breaks (uninterrupted periods of non-use) within a popular commercial PA app called Carrot Rewards over 12 months. We also examined sociodemographic correlates of usage.

**Method:**

This retrospective observational study analyzed data from 41,207 Carrot Rewards users participating in the “Steps” walking program from June/July 2016 to June/July 2017. We measured four usage indicators: duration of use, frequency and length of streaks and breaks, time to first break, and time to resume second streak. We also extracted information regarding participants' age, gender, province, and proxy indicators of socioeconomic status derived from census data. We used descriptive statistics to summarize usage patterns, Kaplan-Meier curves to illustrate the time to first break and time to resume second streak. We used linear regressions and Cox Proportional Hazard regression models to examine sociodemographic correlates of usage.

**Results:**

Over 60% of the participants used Carrot Rewards for ≥6 months and 29% used it for 12 months (mean = 32.59 ± 18.435 weeks). The frequency of streaks and breaks ranged from 1 to 9 (mean = 1.61 ± 1.04 times). The mean streak and break length were 20.22 ± 18.26 and 16.14 ± 15.74 weeks, respectively. The median time to first break was 18 weeks across gender groups and provinces; the median time for participants to resume the second streak was between 12 and 32 weeks. Being female, older, and living in a community with greater post-secondary education levels were associated with increased usage.

**Conclusion:**

This study provides empirical evidence that long-term mHealth app usage is possible. In this context, it was common for users to take breaks and re-engage with Carrot Rewards. When designing and evaluating PA apps, therefore, interventionists should consider the 'multiple-lives' perspective described here, as well as the impact of gender and age.

## Introduction

Participation in regular physical activity (PA) reduces the risk of noncommunicable diseases by improving muscular and cardiorespiratory fitness, functional health, and mental health (1, 2). To obtain these health benefits, it is recommended that adults (aged 18–64 years) participate in at least 150 min/week of moderate to vigorous PA (3). Population-based survey data suggests that over a quarter of adults do not meet PA recommendations globally (4). Finding effective and low-cost strategies to increase PA at a population level remains a public health priority (4, 5).

The proliferation of smartphones including the software applications (apps) that run on these devices offers a promising opportunity for promoting PA at a population level and at a low cost. As of 2021, the global number of smartphone subscriptions (8.6 billion) exceeded the number of people on the planet (7.8 billion) (6). There are more than 3.4 million apps available for download on the Google Play store and 2.2 million for the Apple Play store (7).

Nowadays, people are accustomed to using diverse apps to facilitate their daily lives and support engagement in healthy behaviors such as PA. The popularity of health and fitness apps has been growing in the past few years (8). As of the first quarter of 2021, >50,000 Health apps are available in the Google Play store (9). Data from survey studies (10–13) report that at least 40% of smartphone users have downloaded health and fitness apps—monitoring daily PA is the top reason for using health apps (10, 12, 13).

The number of app-based PA interventions has grown considerably in the past decade. These interventions demonstrate small and short-term improvement in PA outcomes (e.g., 750 steps/d over 12 weeks) (14–20). The field generally agrees that the modest and largely unsustained intervention effects may in part be due to insufficient app usage (21, 22). Usage refers to the actual use of the apps (22), a behavioral aspect of engagement (23). It is commonly measured based on frequency (e.g., number of logins), intensity (e.g., number of app features used), time of duration (e.g., number of days between first and last login) and type of use (e.g., reading a post or taking quizzes) (22). To improve the sustainability of app-based PA interventions, researchers and app designers must understand individuals' usage patterns and identify how best to engage participants.

Existing studies demonstrate that sustaining usage of app-based PA interventions has been challenging (21, 24, 25). A recent review (26) found that 43% of users dropped out of app-based health interventions. Other studies (25–30) also frequently observe that users abandon PA apps after a few weeks or months. However, this body of literature has two notable gaps. First, the evidence is generated primarily in controlled settings with short-term follow-up (20, 28). That means long-term usage of app-based PA interventions in the real-world is not yet well understood. Second, most studies that examined PA app usage have adopted a “single lifetime” perspective (31–33) which assumed users are unlikely to return once they have been absent for a defined period (e.g., 2 weeks). Therefore, previous studies focused on measuring usage at a single time-point, such as identifying the timing when users have lost interest in the app and stopped using it (i.e., non-usage attrition) (34).

Notably, researchers in the field of eHealth and wearable technology have proposed a “multiple-lives” perspective (31–33). Emerging evidence suggest that individuals use wearable technologies or PA tracking apps in multiple disjointed intervals in the real-world (31–33). This means that individuals may engage with an app in streaks [i.e., an uninterrupted series of use days (33)], take breaks [i.e., an uninterrupted series of non-use days (33)], and then re-engage. To our knowledge, only one study (35) has adopted this “multiple-lives” perspective to analyze usage patterns in an incentive-based PA app. Lim et al. (35) examined app usage data from 140,000 individuals who participated in Singapore's National Step Challenge over 7 months. The study found >80% of the participants took more than one break (range: 2.8–10.6), indicating that it is common for users to take breaks and re-engage with an app.

Although several studies (31–33, 35) have verified the 'multiple-lives' usage patterns in PA app users, they focused on identifying the frequency and length of streaks and breaks. While informative, this information is insufficient to inform the design of future apps and engagement strategies. An essential next step is to understand the timing of when streaks and breaks occur and their correlates. To date, these aspects have not yet been examined.

Data from the Carrot Rewards app (Carrot app) provides an opportunity to explore this research question. The Carrot app is one of the very few PA apps that was implemented and rigorously evaluated in a real-world setting (36–39). It is a mobile app that allows users to complete health questionnaires and track steps in exchange for reward points. The Carrot app has demonstrated effectiveness in improving mean weekly daily step counts in a 12-month quasi-experimental study involving over 39,000 users. A positive relationship was observed between the duration of app use and PA outcomes. The intervention effects were more evident for participants who engaged with the app for at least 6 months (36).

In this study, we leveraged the rich usage data from the Carrot app to further investigate how users used the app in the real-world setting over 12 months using a “multiple lives” perspective. The objectives of this study were two-fold. First, we examined duration of use, the frequency, length, and timing of streaks and breaks) within the Carrot app over 12 months. Second, we examined sociodemographic correlates of usage.

## Methods

### The Carrot Rewards app: “Steps” program

Information regarding the theoretical background, evolution and effectiveness of the Carrot app has been published previously (36–38). Briefly, the Carrot app was created by a private company with support from the Public Health Agency of Canada (39). It combined gamification elements (e.g., points, goals, challenges, collaboration and competition) and principles from behavioral economics to engage users and promote physical activity. Users tracked their daily steps and took quizzes on diet, fitness, and personal finance topics to earn loyalty reward points redeemable for consumer goods for programs such as Cineplex's Scene (i.e., movies/cinema), Aeroplan (i.e., air travel), and Petro-Points (i.e., gas/petrol). The app was made freely available to British Columbia (BC) and Newfoundland and Labrador (NL) residents on Apple iTunes and Google Play app stores on 3 March and 13 June 13 2016, respectively. Once enrolled in the Steps program users were instructed to carry their smartphones or wear their *Fitbit* devices during a two-week baseline period to assess habitual PA behavior and set an individualized daily step goal. After the baseline period, users could begin to earn daily incentives ($0.04 CAD) for reaching their step target. After 4 weeks of earning daily rewards, users could then enter a “Step Up Challenge” to earn a $0.40 CAD bonus for reaching their daily goal 10 or more non-consecutive times in 14 days. For users who completed a “Step Up Challenge,” a new higher daily step goal was provided. For unsuccessful users, the previous goal persisted. Participants could earn a maximum of $25.00 CAD in reward points over 12 months. Carrot Rewards was discontinued in June 2019 due to a lack of funding (40).

### Data extraction

We analyzed retrospective data collected from 41,207 Carrot “Steps” program users who enabled the “Steps” walking program (i.e., allowing the app to access their step data) from 13 June to 10 July 2016, and followed them for 12 months. App usage data were automatically recorded daily while using the Steps program. We aggregated daily steps into weekly mean daily steps for each study week. We chose to use average steps by week because our focus was not to examine whether or how streak/break behaviors varied between days but to examine usage patterns over a year. In our previous publications (36, 37), we learned that it was difficulty to identify patterns using daily step or usage data. Aggregating the daily steps into weeks allows us to consider usage behaviors in terms of fewer, but higher-level, meaningful patterns. Another reason is that the number of days of available app data ranged from 1 to 7 days each week. There were missing data within a week that would have to be interpolated, which would be similar to taking the average based on number of days of available data in the week. When registering with the Steps program, users provided informed consent for using their data for research purposes. As part of the privacy policy, users were also informed that data collected in the app for reporting purposes would only be done at the aggregate or deidentified level. The University of British Columbia Behavioral Research Ethics Board approved this study (H17-02814).

### Measures

#### Usage

We defined usage as daily step count data logged. Users could log their daily step count data in the Carrot app by synchronizing with the “built-in” smartphone accelerometer. This “sync” occurred each time users opened the app. For each synchronization, the app automatically retrieved daily step data from users' smartphones or other wearable devices from the past 14 days. In this study, we are interested in whether the users have used the app on a given day, which is similar to the concept of non-wear day in wearable technology studies. Therefore, we categorized a week as a “non-use week” (weekly mean daily step counts = 0) or an “active week” (weekly mean daily step counts >0). As Short et al. (22) recommended, we characterized duration and frequency of use by four measures.

1. Duration of use is the total number of active weeks.
2. Frequency and length of streaks and breaks. Currently, there is no standardized cut-point for defining streaks and breaks. For example, Lim et al. (35) used 1 week, Lin et al. (31) used 30, 60, and 90 days, Meyers et al. (33) used ≥2 days. We operationalized a streak as a period that the app recorded non-zero step counts for two or more consecutive weeks without any breaks; a break is a period with zero steps for two or more consecutive weeks. We used the two-week cut-point because it has been commonly used to determine non-usage attrition in eHealth interventions in the PA literature (30, 41, 42).
3. Time to first break is the number of weeks between the first study week and the first break.
4. Time to resume second streak is the number of weeks between the first break and the week participants re-engaged with the app with at least 2 weeks of non-zero step count.

We focused on the timing for the first break and resuming the second streak because previous studies suggest that users who engaged with the app during the early days or weeks of the intervention appear to predict their adherence to the app (32, 35).

#### Sociodemographic correlates and baseline step count

When registering for the app, participants self-reported their age (years), gender (female, male or others/not specified) and province (BC or NL). We inferred participants' median personal income, percentage of the population with post-secondary education levels, and percentage population identified as visible minorities in their communities by linking user postal codes with census data (i.e., 2011 National Household Survey) at the local health area level (89 in BC) and regional health authority level (4 in NL).

### Statistical analysis

Statistical analysis was performed using R 3.3.0.68 Mavericks build (7202) R Studio Version 1.0.136. For descriptive statistics, we calculated total counts and percentages for categorical variables, means and standard deviations for continuous variables. We plotted illustrative graphs to explore potential usage patterns to examine usage status (active or non-use) for each study week using several random subsamples. Based on visual assessment, we first categorized users who used the app for all 52 weeks in one group and those who had never used the app in another group. Then, we ranked the remaining users based on the number of active weeks and evenly divided them into four groups. The six usage groups were: committed users (52 weeks), frequent users (41–51 weeks), regular users (24–40 weeks), occasional users (11–23 weeks), limited users (1–10 weeks), and non-users (0 week).

We plotted Kaplan-Meier (KM) curves to illustrate the time to the first break and the time to resume the second streak for the total sample and by gender and province. We plotted the KM curves by gender as it was a significant predictor of health app usage in previous studies (43–45) and by province because the two provinces are different in terms of geographic locations, population, weather, and the socio-demographic variables measured in this study.

#### Correlates of duration of use

We fitted two linear regression models that treated duration of use and number of streaks as continuous outcomes. Both models included sociodemographic variables and baseline daily step count mean as predictors. The estimated effects, 95% confidence intervals and Chi-square significance tests were determined for each predictor variable. Given the sample size for the fitted models was large, the magnitudes of the coefficients (i.e., how far they deviated from the null values: 0 for regression estimates and 1 for hazard ratios) and the range of the confidence intervals, were considered for determining practical relevance.

#### Correlates of time to first break and time to resume second streak

We fitted two Cox Proportional Hazard regression models. The first analysis modeled the risk of having the first break at a given week. The time variable was the number of weeks until the first break. The event variable was coded 1 if the first break occurred or 0 if it did not occur. The second analysis modeled the probability of resuming the second streak at any given week after the first break. The time variable was the number of weeks from the first break until the participants resumed usage with a non-zero step count for at least two consecutive weeks. The event variable was coded 1 if resumption occurred or 0 if not. In both analyses, we included the demographic variables and baseline steps as predictors. The hazard ratios [HR], 95% confidence intervals and Chi-square significance tests were determined for each predictor variable. Given the sample size was large, the magnitude of the hazard ratios was considered for determining practical relevance. Due to multiple statistical tests, we did a conservative Bonferroni adjustment to the significance level of 0.05. We set the significance level at *p* < 0.001.

## Results

### Duration of use

**Figure 1:**
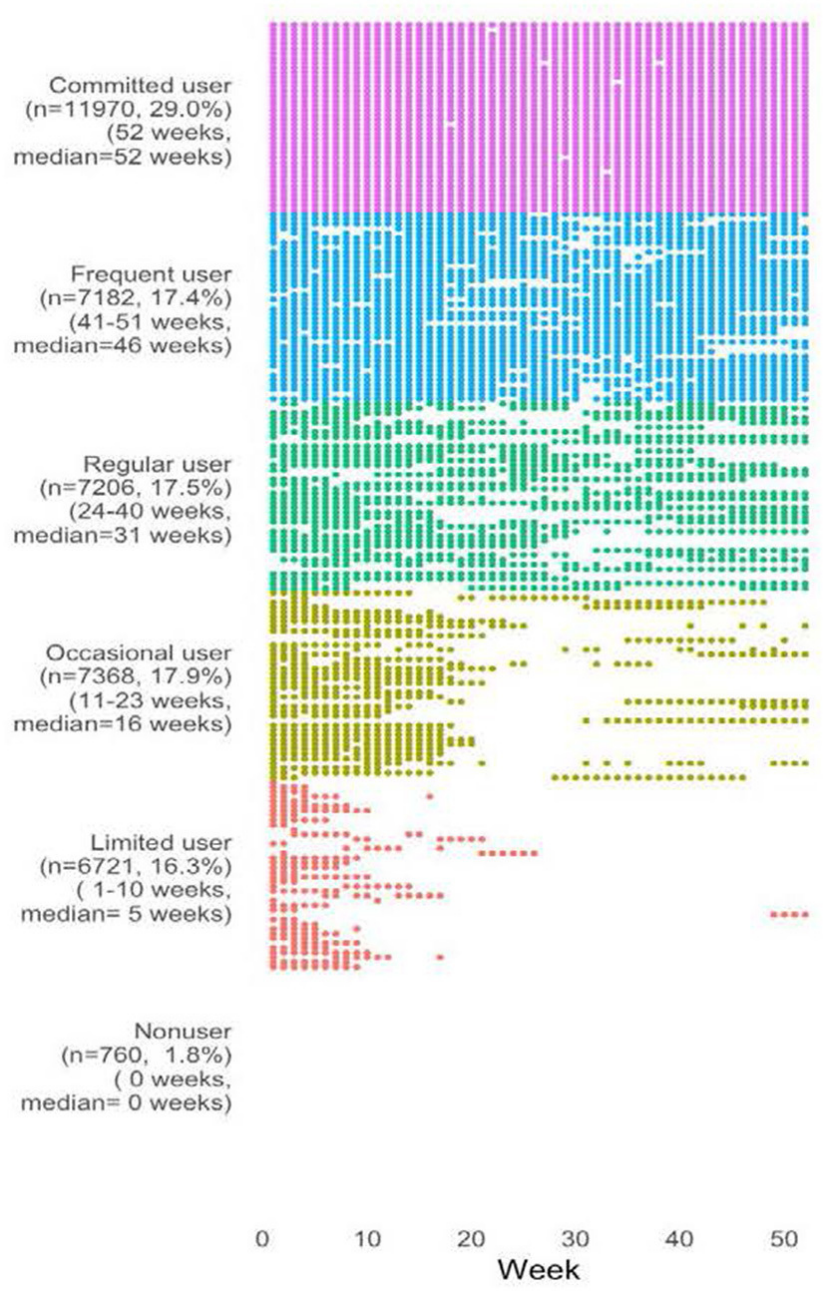
Weeks in use by usage group. Forty users are randomly sampled from each usage group. The plot shows the actual weeks in use.

The final analytical sample included 41,207 users with a mean age of 35.2 ± 11.7 years. Among the users, 67% identified as female, 75% lived in BC; personal median annual income of $29,503 ± 3,997, 55% with postsecondary education, and 26% of the population identifying as a visible minority. The mean baseline daily step counts were 5,537 ± 2,691. Participants used the app for an average of 32.59 ± 18.435 weeks. We illustrate participants' usage in Figure 1. There were 29% committed users who used the app for 52 weeks, 17% frequent users, 18% regular users weeks), 18% occasional users, 16% limited users, and 2% non-users.

### Frequency and length of streaks

The frequency and length of streaks are presented in Table 1. Approximately 98% (40,447/41,207) of the users experienced at least 1 streak. Of those, 60% of the participants had 1 streak and 40% had ≥2 streaks. Participants who had fewer streaks used the app for a longer time. The frequency of streaks ranged from 1 to 9 times (mean = 1.61 ± 1.04 times). The mean streak length was 20.22 ± 18.26 weeks, with a range between 2 and 34 weeks. The mean streak length for each subsequent streak ranged between 10 and 33 weeks for users who had 1 to 3 streaks; between 5 and 10 weeks among users who had 4 to 9 streaks.

**Table 1:** Frequency and length of streaks and breaks.

**Number of streak**	**Number of users**	**Total active weeks** **Mean (SD)**	**S1**	**B1**	**S2**	**B2**	**S3**	**B3**	**S4**	**B4**	**S5**	**B5**	**S6**	**B6**	**S7**	**B7**	**S8**	**B8**	**S9**	**B9**
1	24,920	33 (20.2)	34 (20.2)	35 (12.5)																
2	9,310	33 (14.7)	16 (11.2)	9 (9.3)	18 (12.7)	23 (13)														
3	3,536	31 (12.5)	11 (9.6)	5 (5.2)	9 (8.6)	7 (6.5)	12 (9.2)	17 (11)												
4	1,663	29 (10.9)	9 (7.8)	4 (3.9)	7 (6.9)	5 (4.2)	6 (5.6)	6 (5.3)	9 (7.2)	13 (9.1)										
5	680	28 (9.5)	7 (6.3)	4 (3.3)	6 (5.3)	4 (3.3)	5 (4.6)	5 (3.7)	4 (4.2)	5 (4.3)	8 (6.6)	9 (7.2)								
6	231	25 (8.1)	6 (5.1)	4 (2.9)	5 (4.5)	4 (2.5)	4 (3.5)	4 (2.5)	4 (3.3)	4 (3.1)	4 (3.1)	5 (3.3)	6 (6.1)	7 (5.8)						
7	81	23 (7)	4 (4.0)	4 (1.9)	4 (3.0)	4 (2.4)	4 (3)	4 (2.1)	3 (2.5)	4 (2.5)	3 (2.1)	5 (3.2)	3 (3.9)	4 (2.8)	5 (5.4)	6 (5.5)				
8	21	21 (6.9)	3 (3.2)	3 (1.4)	3 (3.2)	3 (1.6)	3 (1.7)	3 (2.1)	3 (2.0)	4 (2.4)	2 (1.8)	4 (2.4)	2 (1.4)	5 (2.5)	2 (1.6)	4 (2.8)	5 (5.0)	4 (3.3)		
9	5	22 (6.7)	2 (0.9)	3 (0.5)	3 (2.1)	3 (1.6)	2 (1.1)	4 (2.2)	2 (1.9)	3 (1.0)	3 (4.4)	4 (3.2)	3 (2.9)	3 (1.1)	2 (1.1)	4 (2.4)	3 (2.3)	3 (1.3)	3 (1.5)	2 (1.4)

S, Streak; B, Break.

### Time to the first break

**Figure 2:**
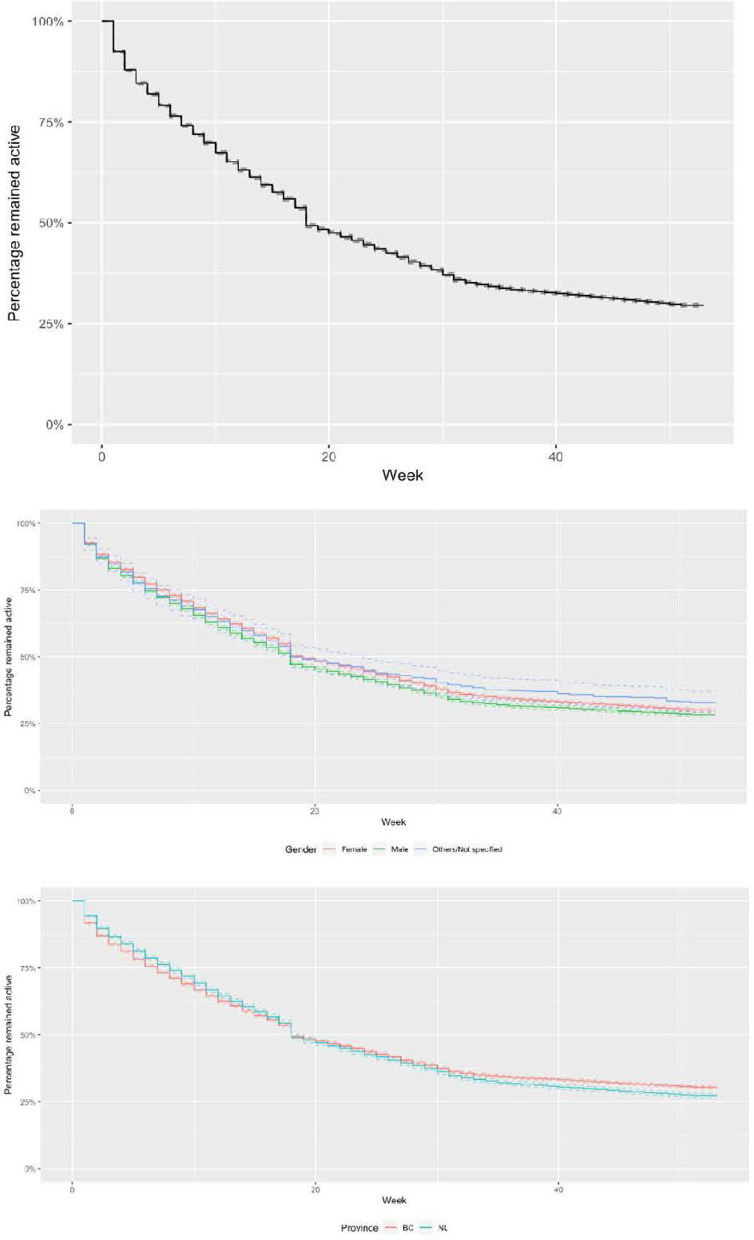
Kaplan-Meier estimates of the survival distribution for time to the first break for the total sample **(top)** by gender **(middle)** and by province **(bottom)**.

The frequency of breaks ranged from 1 to 9 times (mean = 1.61 ± 1.04 times). The mean break length was 16.14 ± 15.74 weeks with a range between 2 and 35 weeks (Table 1). Of users who experienced the first break, the estimated median time to the first break (i.e., the time after which 50% of users experienced the first break) was 18 weeks for the total sample and there was no observable difference between gender groups and provinces. Eighteen weeks for females, 17 weeks for males and 18 weeks for other/not specified gender (Figure 2). As participants began using the app in June/July, their first breaks occurred around November/December (Winter months). The average daily temperature was 5.7/−3.5°C for BC and 2.8/−6.0°C for NL (46).

### Time to resume the second streak

**Figure 3:**
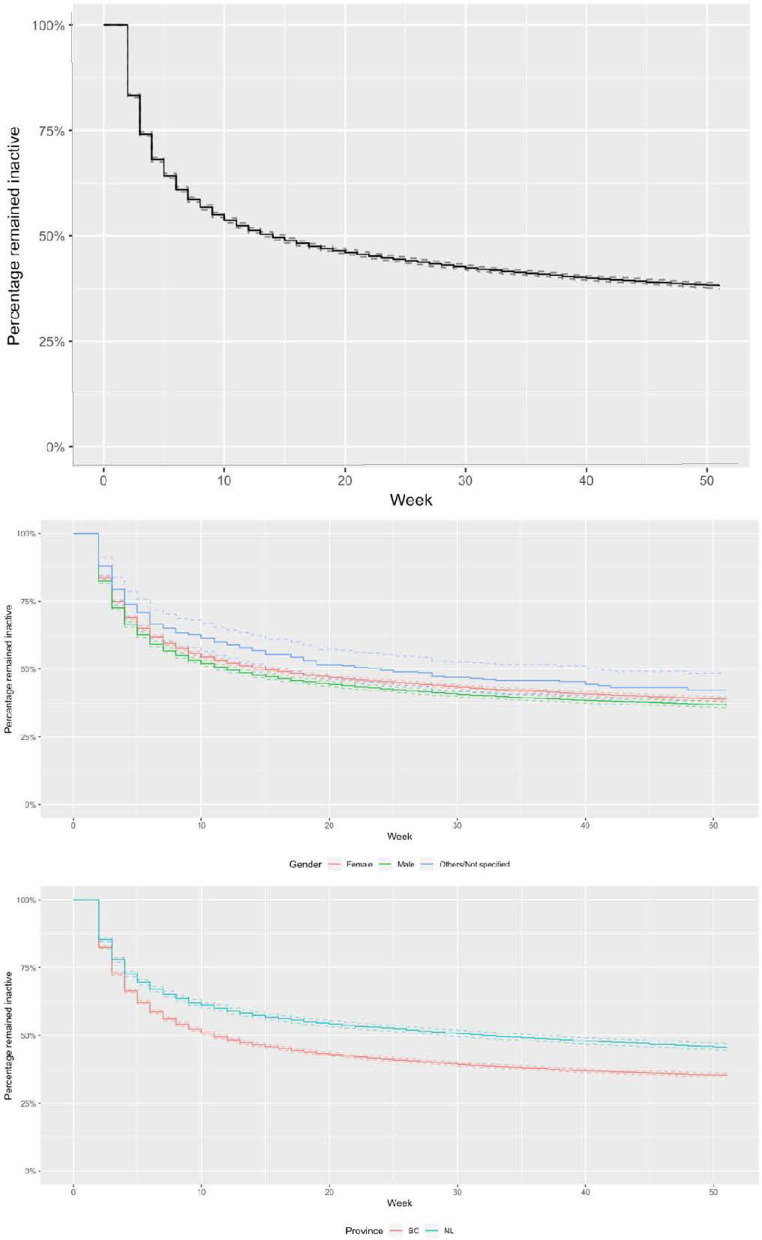
Kaplan-Meier estimates of the survival distribution for time to resume a second streak for the total sample **(top)** by gender **(middle)**, and by province **(bottom)**.

Of users who experienced the first break, 58% (16,506/28,271) resumed the second streak, and 42% did not return. The median time for participants to return for the second streak was 15 weeks for the total sample, 15 weeks for females, 12 weeks for males and 23 weeks for participants in other/not specified gender. The median time to resume the second streak was 11 weeks for BC participants and 32 weeks for NL participants (Figure 3). As participants took their first breaks around November/December, the median time for resuming the second streak occurred in February for BC and August for NL. The difference in the timing to resume the second streak between the two provinces appeared to depend on weather. For BC, the coldest months are between December (−6°C) and February (−1.2°C). For NL, the coldest months typically begins in December (−6°C) until early May (−0.5°C) with late-lying snow patches persisting until July/August in some areas (46).

### Correlates of the duration of use

**Figure 4:**
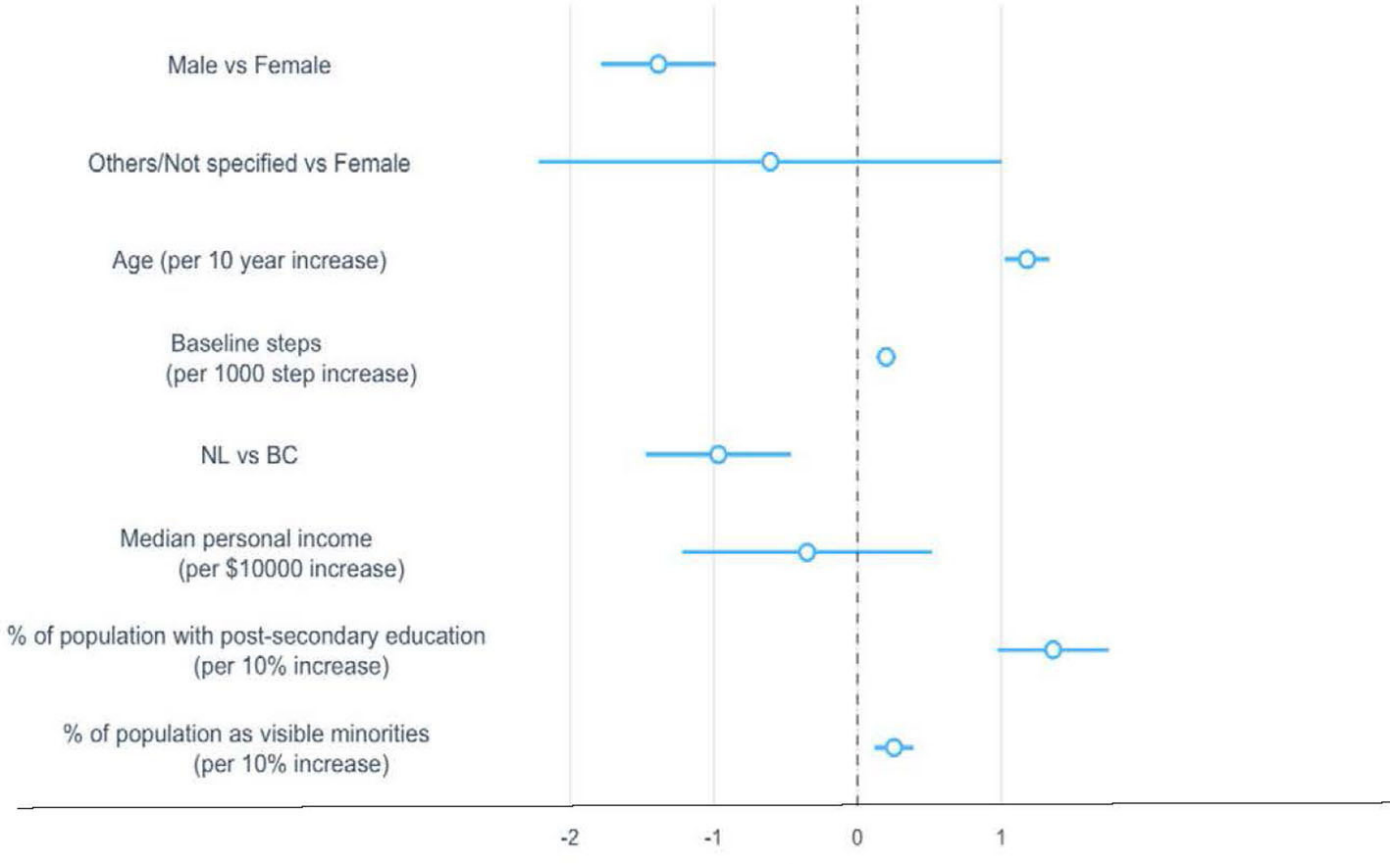
Regression coefficients and 95% confidence intervals for sociodemographic correlates associated with duration of use. The dotted vertical line indicates a mean difference of zero, which means a correlate neither increased nor decreased the duration of use. BC, British Columbia; NL, Newfoundland and Labrador.

Ages, the percentage of population with post-secondary education, and the percentage of population identifying as a visible minority in the community were significant correlates of the duration of use. Gender, baseline steps and provinces had a significant but minimal effect on the average duration of app use. Median personal income was not significantly associated with the duration of use (Figure 4).

Duration of use increased by 1.2 weeks (95% CI: 1.03, 1.34) for every 10-year increase in age, 13.6 weeks (95% CI: 9.74, 17.49) for any 10% increase in the percentage of population with post-secondary education in the community, and 2.5 weeks (95% CI: 1.18, 3.91) for any 10% increase in the percentage of population as a visible minority in the community (Table 2).

**Table 2:** Regression coefficients for sociodemographic correlates associated with duration of use and frequency of streaks.

	**Duration of use**	**Frequency of streaks**
	**Coefficient (95% confidence interval)**	**Coefficient (95% confidence interval)**
Intercept	21.80 (19.78, 23.82)	2.14 (2.02, 2.26)
Gender		
Female	REF	REF
Male	−1.39 (−1.78, −0.99)^*^	0.06 (0.03, 0.08)^*^
Others/Not specified	−0.61 (−2.22, 1.01)	−0.12 (−0.21, −0.25)^*^
Age (per 10-year increase)	1.18 (1.03, 1.34)^*^	−0.01 (−0.02, 0.00)
Baseline steps (per 1000 step increase)	0.20 (0.13, 0.03)^*^	−0.02 (−0.02, −0.01)^*^
Province		
British Columbia	REF	REF
Newfoundland	−0.97 (−1.47, −0.46)^*^	−0.12 (−0.15, −0.09)^*^
Median personal income (per $10000 increase)	−0.35 (−1.22, 0.52)	−0.10 (−0.15, −0.05)^*^
% of population with post-secondary education (per 10% increase)	13.62 (9.74, 17.49)^*^	−0.13 (−0.35, 0.09)
% of population as visible minorities (per 10% increase)	2.55 (1.18, 3.91)^*^	−0.07 (−0.15, 0.01)

* p < 0.001.

### Correlates of the frequency of streaks

**Figure 5:**
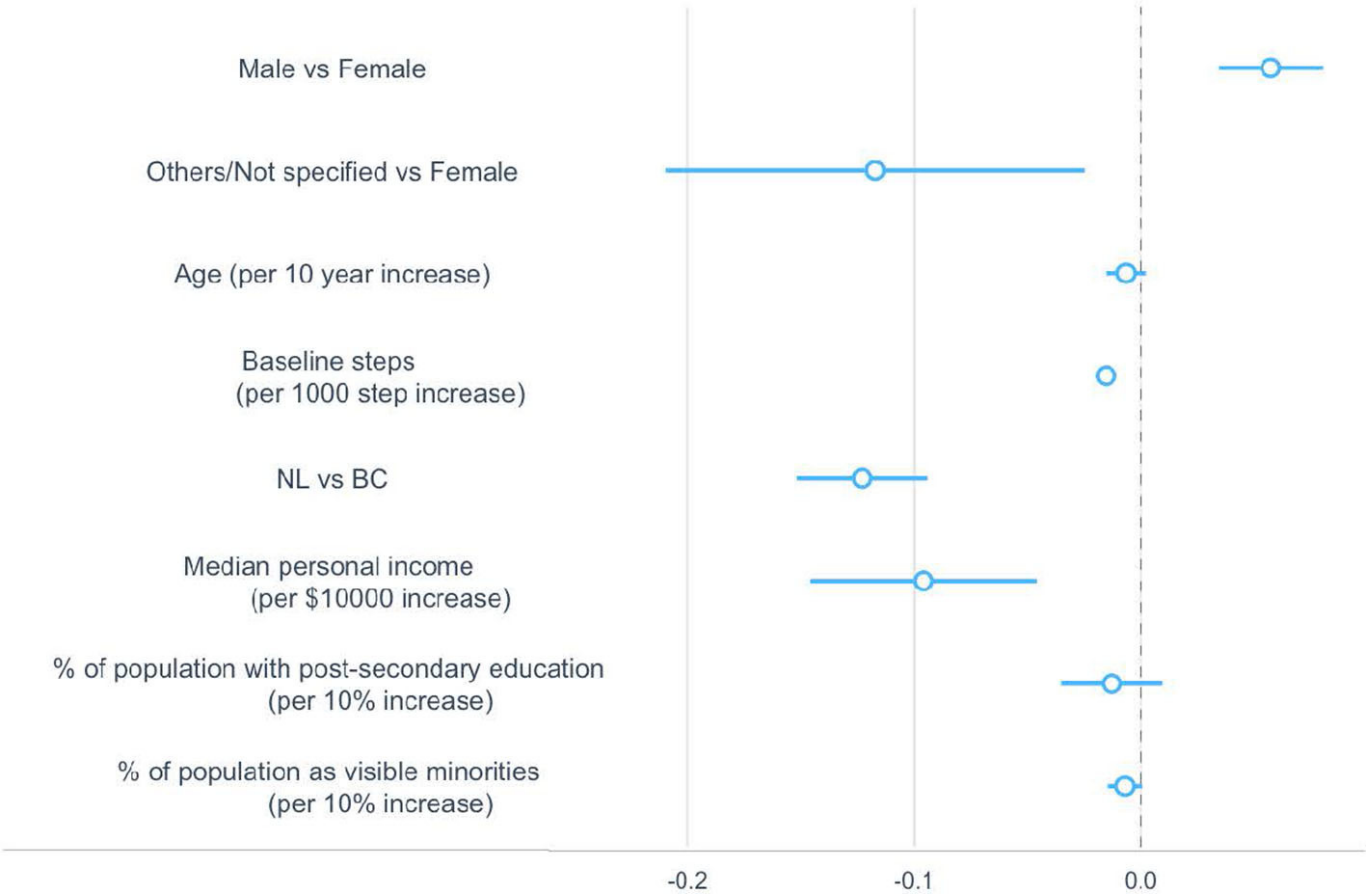
Regression coefficients and 95% confidence intervals for sociodemographic correlates associated with the number of streaks. The dotted vertical line indicates a mean difference of zero, which means a correlate neither increased nor decreased the number of streaks. BC, British Columbia; NL, Newfoundland and Labrador.

Baseline steps, gender, median personal income, and provinces had a significant but minimal effect on the number of streaks. Age, percentage of population with post-secondary education in the community, and percentage of population identifying as a visible minority in the community were not significantly associated with the number of streaks (Figure 5). Users who were female (vs. male), NL residents, had higher median personal income, and higher baseline steps had 0.02–0.12 fewer streaks (Table 2).

### Correlates of having the first break

**Figure 6:**
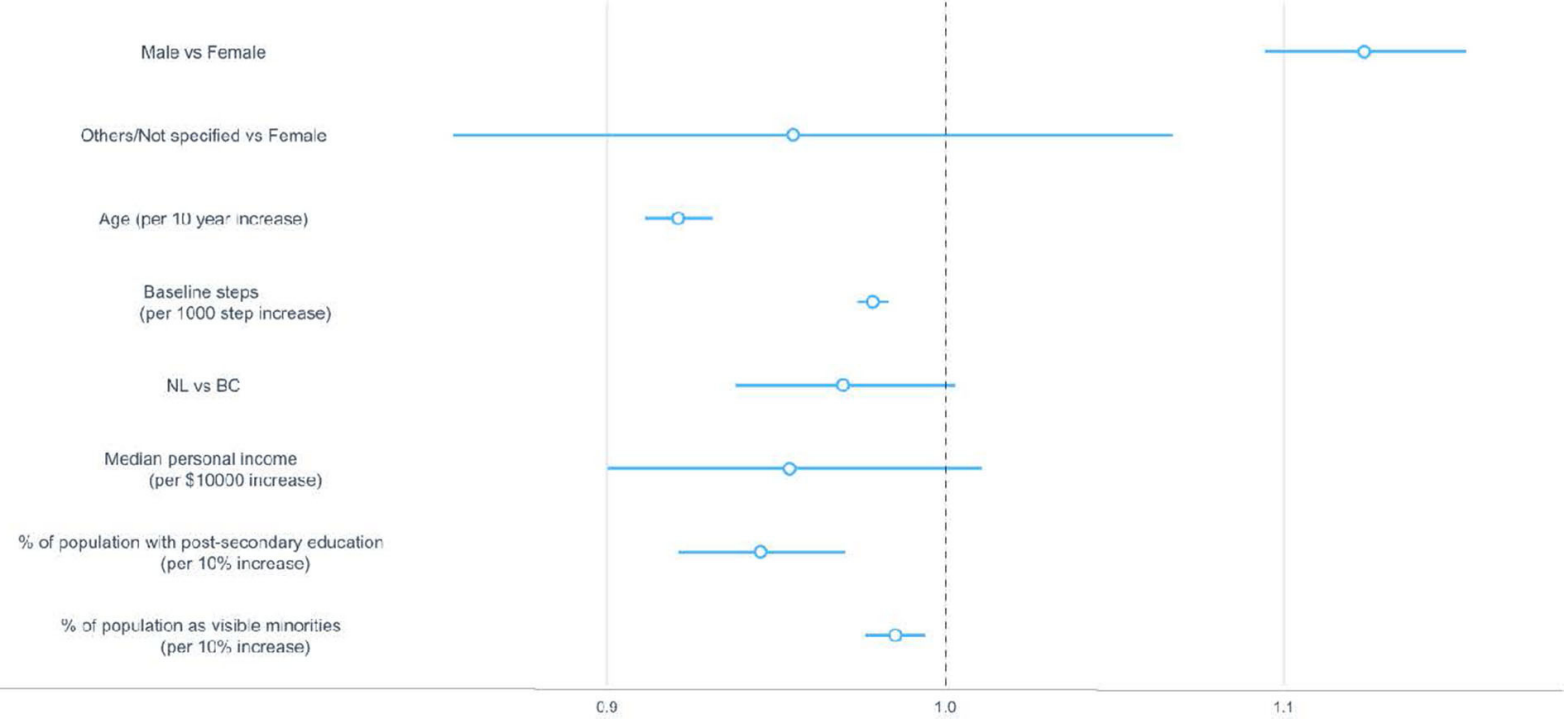
Hazard ratios and 95% confidence intervals for sociodemographic correlates associated with the likelihood of having the first break. The dotted vertical line indicates a hazard ratio of 1, which means the presence of a correlate neither increased nor decreased the likelihood of having the first break. BC, British Columbia; NL, Newfoundland and Labrador.

Age, gender, and the percentage of population with post-secondary education in the community were significant correlates of having the first break. Baseline steps and the percentage of population identifying as a visible minority in the community had a significant but minimal effect on the risk of having the first break. Median personal income and provinces were not significantly associated with the risk of having the first break (Figure 6). Compared to females, males had an average 12% higher risk of having the first break (95% CI: 1.09–1.15). The risk of having the first break reduced by 8% (95% CI: 0.91, 0.93) for every 10-year increase in age, 5% (95% CI: 0.92, 0.97) for any 10% increase in the percentage of population with post-secondary education in the community (Table 3).

**Table 3:** Hazard ratios for sociodemographic correlates associated with the likelihoods of having time to the first break and resuming the second streak.

	**Likelihood of having the first break**	**Likelihood of resuming the second streak**
	**Hazard ratio (95% confidence interval)**	**Hazard ratio (95% confidence interval)**
Gender		
Female	REF	REF
Male	1.12 (1.09, 1.15)^*^	1.06 (1.03, 1.10)^*^
Others/Not specified	0.96 (0.85, 1.07)	0.83 (0.71, 0.96)^*^
Age (per 10-year increase)	0.92 (0.91, 0.93)^*^	1.05 (1.03, 1.06)^*^
Baseline steps (per 1000 step increase)	0.98 (0.97, 0.98)^*^	0.98 (0.98, 0.99)^*^
Province		
British Columbia	REF	REF
Newfoundland	0.97 (0.94, 1.00)	0.77 (0.73, 0.80)^*^
Median personal income (per $10000 increase)	0.95 (0.90, 1.01)	0.87 (0.80, 0.93)^*^
% of population with post-secondary education (per 10% increase)	0.95 (0.92, 0.97)^*^	1.05 (1.02, 1.09)^*^
% of population as visible minorities (per 10% increase)	0.99 (0.98, 0.10)^*^	1.00 (0.99, 1.01)

* P < 0.001.

### Correlates of resuming the second streak

**Figure 7:**
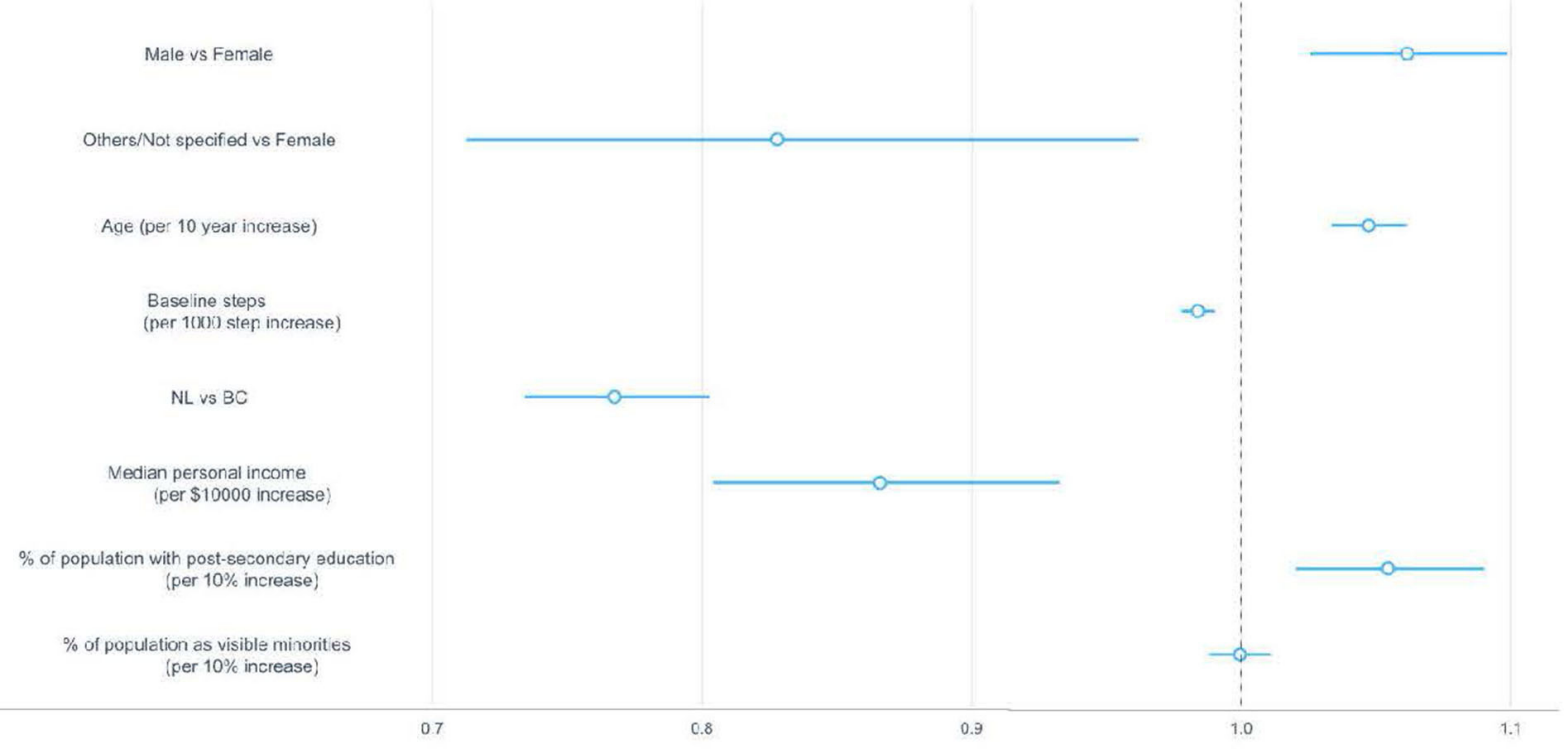
Hazard ratios and 95% confidence intervals for sociodemographic correlates associated with the likelihood of resuming the second streak. The dotted vertical line indicates a hazard ratio of 1, which means the presence of a correlate neither increased nor decreased the likelihood of resuming the second streak. BC, British Columbia; NL, Newfoundland and Labrador.

All the sociodemographic factors were significant correlates of likelihood of returning for the second streak, except for baseline steps and the percentage of population identifying as a visible minority in the community (Figure 7). Compared to females, males were 6% more likely to resume the second streak (95% CI: 1.03–1.10); participants in the other/not specified gender category were 17% less likely to return (95% CI: 0.71, 0.96). NL participants were 23% less likely to return to the app (95% CI 0.73–0.80) compared to BC participants. The likelihood of returning for the second streak increased by 5% (95% CI 1.03–1.06) for a 10-year increase in age, 5% (95% CI 1.02–1.09) for a 10% increase in percentage of population with post-secondary education in the community, and reduced by 13% (95% CI 0.80–0.93) for any $10,000 increase in median personal income (Table 3).

## Discussion

While sustaining the usage of app-based PA intervention is challenging, this study provided empirical evidence demonstrating that long-term usage is feasible. Over 60% of the participants used the Carrot app for more than 6 months, and 29% used it for 12 months. Our findings compared favorably to the 10,000 Steps Australian program, with 0.09% (21/22,142) using the app for 12 months (30) and 50% of the users using the app for <10 weeks (28, 30), and Singapore's National Step Challenge 9% (12,310/139,885) for 6 months and 7% for 12 months (35). We attribute the favorable findings to a synergy of several features of the Carrot Rewards Step program. First, the Carrot app used gamified features including points, goals, challenges, peer leaderboard, teamwork and competition (40). Systematic reviews and a recent randomized controlled trial have found that gamified features enhanced health app usage (29, 47–49). Second, immediate rewards leverage a behavioral economics principle called “present bias” which states that people tend to put more worth and be more satisfied in immediate rewards than with delayed ones. Third, the app required little cognitive effort to use. Once registered, steps were seamlessly tracked and rewarded using the built-in accelerometer so long as the data was synced at least once every 2 weeks. Based on technology adoption theories (50, 51) and empirical studies (52), both the instant reward and ease of use were likely drivers of users' intention to use the Carrot app, which in turn supported sustained use.

Our findings support the “multiple-lives” usage pattern that emerges from wearable technology usage research (31, 33, 35). We observed two types of usage patterns: 60% of the participants had a “single lifetime” pattern (one streak) and 38% had a “multiple-lives” pattern (two or more streaks). A multiple-life usage pattern means that users used, dis-engaged and re-engaged with the app multiple times. In our study, this cycle repeated between 2 to 9 times. This finding has practical implications for designing and evaluating app-based PA interventions in the real world. Future studies should leverage the capability of mobile apps in collecting real-time usage data and utilize such data to conduct streak-and-break analysis. Then, identify critical time points (i.e., weeks before first break, or during first break to re-engage) and develop a bundle of engagement strategies to re-gain users' attention before they abandon the app (e.g., peer support, game-like and flashy features, feature upgrades, bonus incentives, new/fresh app aesthetics (53–56).

Among participants who had a “multiple-lives” pattern, their streak-and-break behaviors appeared to be affected by seasonality. Participants began using the app in June/July, had the first break around 18 weeks (November/December) and returned for the second streak after 23 weeks (March/April) for BC participants or 32 weeks (July/August) for NL participants. This finding suggests that researchers and designers of app-based PA interventions should consider seasonal variations in the analysis and modeling of app usage behaviors and the development and introduction of engagement strategies. In addition to seasonality, other factors may have influenced participants' streak-and-break decisions, such as loss of interest, life interruptions (e.g., pregnancy, new job situation) or no longer needing the app because of successful habit formation (12, 57). Attig et al. (57) found that users who stopped using an app due to loss of interest and successful habit formation were less likely to resume usage. Future studies are suggested to incorporate periodic user surveys and ecological momentary assessments to explore the reasons for abandoning, taking breaks and re-engaging with the app, and the contexts in which users make these decisions (22).

Our results revealed a significant gender difference in app usage. Compared to male, females were more likely to use the app for a longer duration and have lower risks of having the first break. However, females are less likely to return after they take a break. These findings suggest that females are more likely to sustain their initial app adoption and abandonment decisions. Our results are supported by prior research identifying gender differences in health-related internet and app usage (11, 43, 58). Women may be more likely to adopt an app due to its concept while male may be more focused on the functionality of apps (43). These preferences may then somehow influence the sustainability of app usage. While reasons for the gender differences remain speculative the findings do underline a need to take gender into account when developing health apps to ensure they meet the needs and preferences of individuals (58).

Evidence on the socioeconomic correlates of app usage was mixed. Our results demonstrated that older adults and living in a community with a higher percentage of population with post-secondary education (a proxy indicator of socioeconomic status) were associated with increased usage. Yang and Koenigstorfer (50) synthesized findings from 24 studies and found that usage increased with age and income levels. Pontin et al. (44) examined sociodemographic determinants that influenced usage of a commercial incentive-based physical activity app (Bounts) in over 30,000 users over a year. They found that usage was higher in older individuals and users who lived in areas with low socioeconomic status. Carroll et al. (45) found that younger users and those with higher education levels and income use health apps more. The difference in findings may be due to the heterogeneity in the app characteristics, definitions of usage and measures of the sociodemographic variables, and other confounding factors, such as intention to change physical activity behaviors and health status (45).

### Strengths and limitations

Strengths of this study include the large sample size from two Canadian provinces, long follow-up period, the inclusion of objective measurement of app usage collected in a real-world setting. Our streak-and-break analysis is innovative and offers insightful perspectives on the everyday use of a commercial PA app. Our findings on a multiple-life usage pattern extend the literature in PA apps by providing an example of modeling streak-and-break behaviors, identifying re-engagement time-points and corresponding strategies to increase re-engagement.

The findings presented here should be interpreted in the context of the study's limitations. We determined usage patterns based on aggregated weekly data, which could result in very different usage patterns compared to studies using daily data (33, 35). We have relied on objective usage metrics from the study's database. This study only focused on duration and frequency of use and did not measure intensity and type of use. Lin et al. (31) found that individuals could engage in different kinds of app features and at different intensities in each lifetime. Metrics on these usage aspects could facilitate our understanding of the psychological mechanisms underlying the streak and break behaviors (59). In this secondary data analysis, our analyses were limited to data collected in the original study design. Therefore, we cannot consider other psycho-social correlates that may explain the variations in usage among individuals, such as self-efficacy or habit strength.

## Conclusion

We demonstrated a real-world example of how individuals used a commercial PA app over 12 months. It is common for users to take breaks and re-engage with an app. Interventionists need to adopt a 'multiple-lives' perspective when designing and evaluating app-based PA interventions. We also need more real-world studies with long-term follow-up to facilitate our understanding of individuals' usage behaviors and inform the development of engagement features of future apps. Gender and age may be significant correlates of long-term usage of app-based PA interventions.

## Data availability statement

The data analyzed in this study is subject to the following licenses/restrictions: The datasets used and/or analyzed during the current study are available from the corresponding author on reasonable request. Requests to access these datasets should be directed to erica.lau@ubc.ca.

## Ethics statement

The studies involving human participants were reviewed and approved by University of British Columbia. Written informed consent for participation was not required for this study in accordance with the national legislation and the institutional requirements.

## Author contributions

MM collected and prepared the data. EL and GF performed data analysis. EL wrote the first draft of the manuscript. All authors commented on previous versions of the manuscript, contributed to the study conception, design, read, approved the submitted manuscript, and have agreed to be personally accountable for their contribution.

## Funding

GF and EL were supported by a Canadian Institutes of Health Research/Public Health Agency of Canada Chair in Applied Public Health Award to GF (GR012882). EL is supported by the Michael Smith Health Research BC Research Trainee Award (GR018656).

## Conflict of interest

Author MM received consulting fees from Carrot Insights Inc. from 2015 to 2018 as well as travel re-imbursement in January and March 2019. He had stock options in the company as well, but these are now void since Carrot Insights Inc. went out of business in June 2019. The remaining authors declare that the research was conducted in the absence of any commercial or financial relationships that could be construed as a potential conflict of interest.

## Publisher's note

All claims expressed in this article are solely those of the authors and do not necessarily represent those of their affiliated organizations, or those of the publisher, the editors and the reviewers. Any product that may be evaluated in this article, or claim that may be made by its manufacturer, is not guaranteed or endorsed by the publisher.
